# Rwandan family medicine residents expanding their training into South Africa: the use of South-South medical electives in enhancing learning experiences

**DOI:** 10.1186/s12909-015-0405-3

**Published:** 2015-08-01

**Authors:** Maaike Flinkenflögel, Gboyega Ogunbanjo, Vincent Kalumire Cubaka, Jan De Maeseneer

**Affiliations:** 1 Discipline Primary Health Care, College of Medicine and Health Sciences, University of Rwanda, Kigali, Rwanda; 2 Partners in Health, Rwinkwavu, Rwanda; 3 Department of Family Medicine and Primary Health Care, Ghent University, Ghent, Belgium; 4 Department of Family Medicine & Primary Health Care, Sefako Makgatho Health Sciences University, South Africa (previously known as University of Limpopo – Medunsa Campus, South Africa), Limpopo, South Africa; 5 Center for Global Health, Department of Public Health, Aarhus, Denmark

**Keywords:** International medical electives, Global electives, Global health, Medical education, Learning outcomes, Family medicine training, South-South, Africa, Ethics

## Abstract

**Background:**

International medical electives are well-accepted in medical education, with the flow of students generally being North–South. In this article we explore the learning outcomes of Rwandan family medicine residents who completed their final year elective in South Africa. We compare the learning outcomes of this South-South elective to those of North–South electives from the literature.

**Methods:**

In-depth interviews were conducted with Rwandan postgraduate family medicine residents who completed a 4-week elective in South Africa during their final year of training. The interviews were thematically analysed in an inductive way.

**Results:**

The residents reported important learning outcomes in four overarching domains namely: medical, organisational, educational, and personal.

**Conclusions:**

The learning outcomes of the residents in this South-South elective had substantial similarities to findings in literature on learning outcomes of students from the North undertaking electives in the Southern hemisphere.

Electives are a useful learning tool, both for Northern students, and students from universities in the South. A reciprocity-framework is needed to increase mutual benefits for Southern universities when students from the North come for electives. We suggest further research on the possibility of supporting South-South electives by Northern colleagues.

## Background

International medical electives (IME) are a well-established part of medical curricula globally. Universities invest in students going abroad as there is educational value in being exposed to a different context and environment with a dissimilar patient-population, an unfamiliar health system, a diverse scope of medical problems and diseases, dissimilar equipment, and trainers with different skills set and approaches [1–6].

The majority of IME described in medical literature focuses on medical students from the “North” (developed countries) going to the “South” (developing countries) to be exposed to the clinical practice, to learn about diseases rarely seen in their region and to experience different cultures [2, 7–9]. Only occasionally does medical literature describe South–North electives [10]. Very little is known or described on IME within Africa. In this article we describe a South-South elective. Rwandan Family and Community Medicine postgraduate residents went to the University of Limpopo in South Africa as a pilot-study to describe learning outcomes in South-South electives.

### International medical electives

Electives are described as study periods where students have the choice of subject, teacher, study method and/or study location. Already two centuries ago it was acknowledged that electives have a positive value to medical education and lead to more broadly educated students [11]. IME exposes students to rare clinical diseases and other health care delivery systems, It improves diagnostic reasoning with little diagnostic tools and clinical skills, it commits to underserved populations or rural areas and increases cultural competence [9]. In addition, IME encourages self-directed learning, personal development and general education [4]. Niemandsverdriet [7] framed the meaningful learning outcomes in six domains namely: medical knowledge, skills, international health care organisation, international medical education, society and culture, and finally personal growth. A literature review by Dowell and Merrylees [12] divided the key learning of medical electives into 4 main domains namely: clinical skills/knowledge, attitude, global perspectives, and personal/professional development, with two broader benefits: institutional benefits and moral/ethical considerations.

Overall it is agreed that medical electives have a positive impact on the education of students. Though Kumwenda et al. [5] argued that a relevant ethical framework is needed to prepare students better for the experiences they will encounter. Recently, authors have been discussing that North–South IME should not only gain students, but also profit the hosting institutions/communities. Mutual benefit is of utmost importance and it is granted that IME should not be exploiting the host institutions [13, 14]. Gupta and Farmer [15] argued that going abroad shouldn’t be an “elective safari” but in exchange for the educational experience, students should return something in a wise, responsible, and ethical way benefitting the patient and community they worked with. Several authors have since argued for reciprocity in IME. Though, how to address this has been complicated. Bozinoff et al. [16] interviewed hosting supervisors and drew the conclusion that mutual benefit is essential for future collaboration between the various institutions. The Working Group on Ethics Guidelines for Global Health Training (WEIGHT) developed a set of guidelines, including the focus on mutual and reciprocal benefit [14, 17].

### Family medicine training in Africa

Family medicine in South Africa has been part of the university training since the 1960s. The specialty is presently well-integrated in the South African health care system. The South African model for family medicine is distinctive, with academic and service elements based in, and responsible for clinical services in both referral/district hospitals and in community health centres for primary care delivery [18]. Since 1997 South African universities started working together [19]. Later other universities in Sub-Sahara Africa joined and together they have formed a network to improve the development of African family medicine training, called Primafamed [20]. This network has a focus on South-South cooperation between African departments of family medicine and strengthening family medicine training on the African continent. In 2009 a “Statement of Consensus on Family Medicine in Africa” defined the contribution of family medicine to equitable and quality primary health care within the African context, and the role and training requirements of African family physicians [21].

The Rwandan health system presently has medical specialists in the referral hospitals, while district hospitals are run by general practitioners without further academic training after finalizing medical school. The family and community medicine residency (FAMCO) at the National University of Rwanda started in 2008 with the vision to train generalist-specialists based in district hospitals. FAMCO focused on improving quality of care at a lower level, within the concept of the African family physician [21, 22]. The speciality of family medicine was a new concept in Rwanda and is struggling for its continuation. The FAMCO training programme offered the first batch of final year residents the opportunity to undertake an elective at Limpopo University, South Africa, where family medicine was well-established.

## Method

### Research question

The research question we explored was “How does a medical elective in South Africa add value to the Rwandan postgraduate training of family and community medicine as perceived by the residents?”

The aims of the study were to determine what FAMCO residents gained (learning outcomes) from the elective in South Africa and how this satisfied their set objectives and expectations. We compared outcomes of this research with literature on North–South medical electives.

### Research methodology

In this qualitative descriptive research, semi-structured individual interviews were carried out with all five final-year Family and Community Medicine residents from the National University of Rwanda. All these residents experienced a four-week elective at the University of Limpopo, South Africa.

### Data collection

One researcher (MF) performed an audio-taped in-depth semi-structured interview with each of the five residents who completed their elective in South Africa. Consent was obtained from each interviewee and interviews were individually conducted in English. The researcher used open ended questions, reflective listening, summarizing, and elaboration to conduct each interview. The focus was on the resident’s objectives and expectations, experiences and the value of the elective. The electives took place between June and August 2012 and the interviews were held in September 2012. Ethical approval was obtained by the National University of Rwanda Research Ethics Board.

### Data analysis

The data was thematically analysed using Microsoft OneNote. Three hundred and eighty (380) minutes of audio-recorded data were collected and transcribed verbatim. Analysis was done in an inductive way and started from a small existing hypothetical framework (knowledge/skills, SA health care system, family medicine practice, family medicine training) that came up after the transcripts were read for the first time. While going through the data again, this framework was broadened with more clusters and deepened with sub-elements. Finally it was re-organised in 4 main domains while analysing the text line by line. In addition, the second researcher independently reviewed the transcribed data, domains and supporting evidence for the domains to improve the trustworthiness of the study.

## Results

All five interviewees were 4th (final) year Family and Community Medicine residents, Rwandan, males and aged between 30 and 50 years. Four of the residents went in pairs for the elective, the last went alone. The elective was four weeks.

### Objectives and expectations of the elective

The students’ objectives and expectations were grouped into five overall objectives: to increase knowledge and skills in patient care, to learn about the South African health care setting, to learn about the development of family medicine, to learn about the set-up of the postgraduate training program, and finally to extend their network with other family physicians. The following quote gives a general idea of the objective of one of the residents:

*“I wanted to go there to see how our big brothers are doing it in terms of family medicine. Because you know that we are pioneers in terms of family medicine in Rwanda” (R1)*

### Learning outcomes of the elective

Twelve cluster of learning outcomes emerged from the data, which we categorised under four overarching domains: medical, organisational, educational, and personal (Table 1).

**Table 1:** Learning outcomes

1. Medical domain	
a. Medical knowledge	
b. Attitude	
c. Skills	
2. Organisational domain	
a. Health care organisation	
b. Patient Care	
c. Family Medicine	
3. Educational domain	
a. Teaching methodology	
b. Research	
c. Family Medicine training	
4. Personal domain	
a. Networking	
b. Cultural experience	
c. Personal growth	

#### 1. Medical domain

##### Medical knowledge

The residents saw and discussed numerous cases of HIV and TB co-infection and were impressed by the high numbers of infection (*in 2012 HIV prevalence was 2.9 % in Rwanda vs. 17.9 % in South Africa* [23]). Residents thus gained experience and got an opportunity to learn by comparing the South African disease management with their daily practice in Rwanda. The following quote exemplifies this: *“You see that a patient with HIV, if he is coughing, most likely for them they are thinking of TB, …, in Rwanda we say, probably it is bacterial, if the sputum is negative, let’s put the patient on 6, 7 to 14 days of antibiotics, to see if it is really TB, but there in South Africa, no, they cannot wait that long time. They put the patient on anti TB.”* (R1)

They also saw a good number of patients with non-communicable diseases in the OPD/wards. The high number of rape cases they saw surprised them.

##### Attitude

The residents felt the elective improved their attitude around patient care provision in a positive way. It encouraged them as they saw how working with patients in a patient-centered way works better for both patient and doctor. The elective motivated them to improve communication (doctor-patient and inter-professionally).

They experienced there was a strong learning-culture at the training site they were based. This encouraged them in putting more effort in their own study after the hours spent in the clinics. Finally they experienced that family medicine had a well-functioning role in the health system which increased their confidence. Though they felt dispirited at times when they saw their colleagues working with more equipment and living in better circumstances (higher salaries, cars) compared to the Rwandan setting.

##### Skills

Due to the visa restrictions, the residents were merely observers and there were few actual skills they learned during this training. They had hoped to learn more surgical skills which did not happen. A few residents mentioned that a skill they learned was to organise their work. One resident mentioned he learned how to link the theory to practice, specifically when he observed the skills lab as a place to learn procedural and clinical skills.

#### 2. Organisational domain

##### Health care organisation

Free health care with a strong focus on primary health care, fewer referrals, good follow up and continuity of care were mentioned often. Residents felt that sufficient resources (diagnostic, treatment, well-trained/well-paid humans, and infrastructure) in the South African health care system led to high quality care, higher accessibility of services and higher motivation of health care personnel. There were several challenges the residents observed around health care delivery in South Africa. Problems in healthcare-seeking behaviour (despite free and good quality care, patients come to clinics too late) and unhealthy lifestyle (smoking, alcohol consumption, obesity) were mentioned.

##### Patient care

Important elements were the doctor-patient relationship, trust, patient-centred care, and patients’ rights as expressed in this quote: *“Sometimes we don’t give enough attention to the relationship between patient and doctor. I think it is necessary the way you are talking to the patient will determine even the compliance to your treatment. So (you) it’s best for you to not talking ugly to the patient, to not condemn him, not blame on him so that the patient will get more confidence on you”. (R5)*

Residents observed good food-intake by patients, and found no malnutrition in children. Two residents had the theory that due to good food-intake, people’s health was better than in Rwanda, leading to less health problems despite poor ARV-adherence and late consultations. Residents were surprised to find abortions legalized in South Africa, which they found difficult to comprehend.

##### Practice of family medicine

The residents felt they got a better understanding and awareness of the practice of family medicine in Africa as they saw it integrated in the South African health care system. The following positive aspects were recognised: South Africa provides a full support from authorities and family medicine is well integrated in the health care system. Secondly they acknowledged a good organisation of family medicine at all levels of health care. Thirdly they recognised how family medicine provided continuous, comprehensive and cost-effective care. And finally they noticed that Family physicians and registrars are well paid which according to them increases motivation. The following quotes provide evidence for their interpretation of family medicine within the South African context: *“Even the cost-effective because you know the old patients was passing to the family medicine clinic department, the family physician would orient the patient when it is necessary, and they would orient the patient to the right way. Not going to the cardiologist and the cardiologist say it is not my problem, go to the nephrologists and say it is not my business and go to the pneumologue and so on. But as family physician we see the patient is referred to a specialist, you know where exactly you are going to orient him. So family medicine it is a cost-effective department.”(R5)*

#### 3. Educational domain

##### Teaching methodology

The residents named several teaching methods they experienced to improve learning, which they appreciated. These included quality supervision, interactive teaching with stimulating discussions, research mentorship from early in the training, skills lab with manikins, weekly presentations for everyone (from interns to professors), and a big library available for all.

##### Research

The residents experienced a strong research culture at the department with regular presentations and encouragement to work on own research projects or to develop quality improvement projects.

##### Family medicine training

They learned how the family medicine department was running with physicians of different training levels. And they experienced how the family medicine department used the teaching methods and research to increase its output. They were surprised to find family medicine modules incorporated in the undergraduate training.

#### 4. Personal domain

##### Networking

Residents felt they were able to extend their network during their stay. They said now they can contact South African colleagues to share information, work on research or get guidance in further development of family medicine in Rwanda. The latter is supported by the following: *“I can for example have a need to someone that I can ask him information, he can direct me, even once when I am back here in Rwanda, because I have their contact, they know me, we know each other, I can contact them for more information.”(R1)*

##### Cultural experience

Experiencing South Africa as a different culture with different places was seen as a positive added value to the elective as supported by the following quote:

*“it was a good occasion to go there even if, if you leave the part of medicine it is also time to visit time to go. And to see beautiful places and for shopping and to visit”(R2)*

##### Personal growth

The elective increased their confidence and strengthened their belief in reaching difficult goals in both professional and personal life. This elective also gave them the opportunity to reflect on their own country and to see the challenges and opportunities they have in Rwanda. One of the interviewees responded as follows:

*“when I said that it helps me to grow mentally or psychologically, to say that yes, I have a future (…) a family physician is a valuable man”(R5)*

### Reaching the objectives and expectations

Most of the objectives were reached, although not always in depth as was expected, but overall the residents felt the elective added value to their residency training as supported as follows: .

*“I think I reach those objectives because after the elective, I got more clear vision of family medicine, the place of family medicine in the health system and I got more about a clear vision of organization of family medicine, and then I have learnt something that I can implement in our context” (R5)*

The objective that was not reached was to gain better surgical skills. They also expected to gain more medical knowledge, with use of standardized protocols, and to learn about family medicine care in the district hospital setting. Several reasons were mentioned for not reaching these objectives and expectations. The first was time, as 4 weeks was too short to cover all objectives. Secondly the location, the elective was based mainly in the referral hospital, where the focus was on adult cases, with some outreach to health centers, not in the district hospital with a more comprehensive care. Thirdly the language was a barrier as the residents were not familiar with the local language that was often used by patients and care providers. And finally the visa/work permit constrictions made that they were only allowed to shadow and not to provide direct patient care.

### Limitations

The study had a small sample size (5). All came from the same background of final year family and community medicine training in Rwanda going to the same hosting institute (Limpopo). However, as this was a qualitative study, the quality of information obtained was more important than the sample size. IME within the South–South context have not yet been described in literature, therefore this research can be seen as a pilot study.

The elective is part of the postgraduate curriculum. In this study we have been used literature on electives in undergraduate medical training. Literature on electives in postgraduate training (South–North), is usually focussed on specific procedures and skills, whereas this elective was more comprehensive. Consequently, the objectives, expectations and outcomes of this elective seemed to be better comparable with the framework of undergraduate North–South electives.

The interviewer, who also analysed the data is one of the faculty of the department and therefore directly involved in training the residents. This might have caused a reporting bias as the residents might not have answered in a similar way, as they would have with an unknown interviewer. However when analysing the data we believe this was minimal as resident’s responses ranged widely from positive to negative experiences. When the second researcher reviewed the analysed data there was consistency in the interpretation of the data.

Interviews were conducted in English, which for all residents is either their 2nd or 3rd language. This is visible from the grammatical quality of the quotes. However, residents are used to expressing themselves in English every day, and we generally found it easy to understand the meaning of what was said.

## Discussion

Similarities and differences compared to earlier findings in literature on learning outcomes in electives were found. The systematic review from Dowell and Merrylees [12] on learning outcomes and experiences of students in international electives reported four main domains: clinical skills/knowledge, attitude, global perspectives, personal/ professional development: with two broader benefits: institutional benefits and moral/ethical considerations. In our findings we combined knowledge and skills with attitude. In attitude we included residents’ emotional expressions of their own experiences in providing health care, while Dowell explained attitude as awareness of clinical responsibility, public health and resource use. This ‘awareness’ we covered in the clusters of health care organisation and patient care (*Organisational*). Their literature search did not describe what we covered under *Educational*. In our findings the residents did not mention the two broader benefits ‘institutional benefits’ and ‘moral/ethical considerations’. The semi-structured questionnaire we used did not specifically ask this and students did not bring it up themselves. A recent article from Ventres and Wilson [24] focused specifically on the attitudinal goals and traps during what they called international service-learning trips from North to South. They discussed how attitude is of utmost importance in the success of failure of the international experience [24]. Further exploration of how their attitude framework could be extrapolated to IME within Africa would be valuable.

Overall residents were satisfied with how this elective helped them to reach their objectives and expectations. However several circumstances such as little time, visa restrictions and location of elective hindered the accomplishment of all. Issues like this can be discussed and improved with new cohorts of students in future electives, so objectives and expectations can be adapted.

Currently at most Northern universities students are stimulated to do electives outside of their safety zone. A literature review by Cherniak et al. [6] reported that in the USA and UK respectively 30 % and 40 % of medical students are doing IME. These are often done in the South, mainly Africa. However, African medical students rarely get the opportunity to go for an international elective and gain from its positive values. This is bringing up the issue of mutual benefit, as also stated in the Ethics and Best Practice Guidelines for Training Experiences in Global Health (WEIGHT guidelines) [14].

It is important to better frame and analyse the reciprocity of electives, i.e. the benefits as seen from both sides. When bringing students from North to South, how can Southern universities benefit? Supporting Southern students to go to the North for training has been one option, but there are challenges such as high expenses, possible brain drain, learning non-transferable skills and low numbers of students. Similar challenges may be found in the South-South IME, and research is needed to further investigate this. The elective we described was done in one direction from Rwanda to SA. There is a need to explore benefits of electives within Africa in both directions with the aim of generating a reciprocal relationship that could make the electives mutually beneficial and thus more sustainable.

## Conclusion

The elective played an important role in the learning process of the final year Family and Community Medicine residents. Even though the elective had its limitations, the residents all expressed how they saw it as a fruitful experience. It increased their confidence and upon return they felt they were better doctors to serve the Rwandan community, as the elective increased their knowledge and positively influenced their attitude.

IMEs are very common in universities in the developed world, though they are not only useful for Northern students. IMEs can add valuable capacity to the education for students from the South. Southern universities should look into the possibility of developing or improve their networks in the South. Our study hypothesises that teaching sites within Africa, when well organised, may be more practical, well-functioning and potentially cost-effective for electives for African students. Which will provide them with useful knowledge and skills and improve their attitude.

**Fig. 1:**
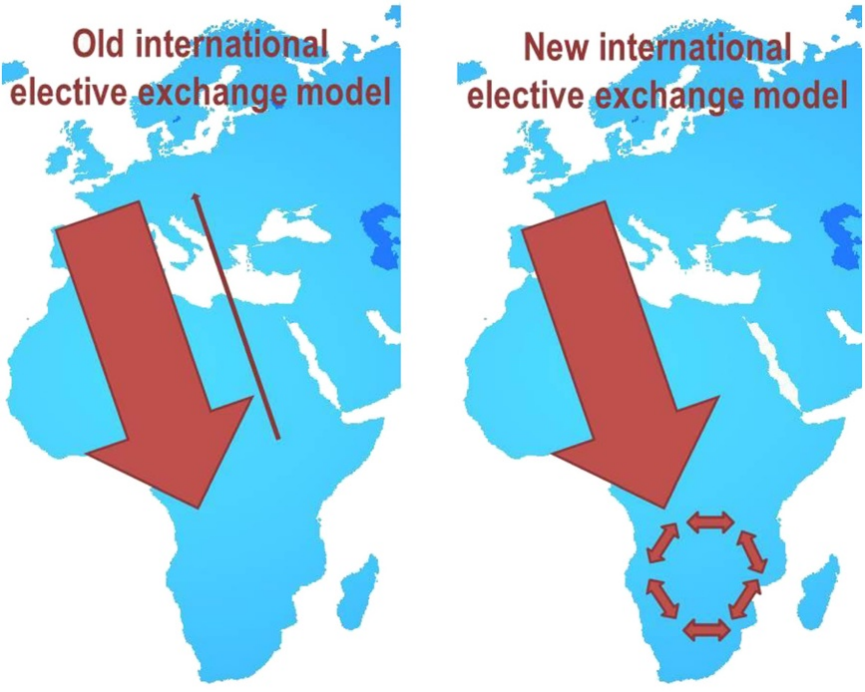
The North–south-South elective exchange model. The North–south-South elective model as a way to strengthen Southern universities and to give more students from the South the possibility to experience IME

The development of North–South-South partnerships, where South-South elective programs are supported by the Northern universities who also send their students to the South (Fig. 1) may be another approach to consider. We see the need for further research to explore the practicality of electives within Africa and models like this suggested N-S-S model. We urge Northern institutes to review their vision and actions for reciprocity when sending students to their Southern colleague institutes.
